# Posterior Reversible Encephalopathy Syndrome After Infliximab Infusion for Fistulizing Crohn's Disease

**DOI:** 10.14309/crj.0000000000001400

**Published:** 2024-06-21

**Authors:** Qusay Abdoh, Razan Rabi, Basel Musmar, Ahmad Abuhassan, Abdulkareem Barqawi

**Affiliations:** 1 Department of Gastroenterology, An-Najah National University Hospital, Nablus, Palestine; 2 Department of Internal Medicine, An-Najah National University Hospital, Nablus, Palestine; 3 Department of Medicine, An-Najah National University, Nablus, Palestine; 4 Department of Neurology, An-Najah National University Hospital, Nablus, Palestine; 5 Department of Surgery, An-Najah National University Hospital, Nablus, Palestine

**Keywords:** Crohn's disease, infliximab, posterior reversible encephalopathy

## Abstract

Biologic therapy is the mainstay of treatment of complicated inflammatory bowel diseases, which has numerous potential side effects. Among these is a rare condition known as posterior reversible encephalopathy syndrome (PRES), which is a reversible neurological disorder that results in symptoms such as headache, nausea/vomiting, blurry vision, and seizure and is diagnosed based on specific clinical and radiological features. This report presents a case of a 19-year-old woman with fistulizing Crohn's disease who was treated with infliximab, but subsequently developed PRES, which was manifested as recurrent episodes of seizures and elevated blood pressure readings, was managed supportively with antiepileptic and antihypertensive medications and eventually made a full recovery, even after resuming infliximab. This case adds to the fewer than 10 previously reported cases of PRES associated with biological therapy for inflammatory bowel disease. It highlights the need to consider this complication when prescribing these drugs.

## INTRODUCTION

Infliximab is a chimeric immunoglobulin G antibody that effectively inhibits tumor necrosis factor (TNF)-α thereby that exhibit potent anti-inflammatory effects.^1^ As a result, it has become a standard treatment option for moderate-to-severe or fistulizing Crohn's disease (CD) that is resistant to standard therapies, used for induction and maintenance.^1^ Although its uses have spread in various fields, there are still concerns about its potential adverse effects, such as infection risk, hypersensitivity, immunosuppression, immunogenicity, and carcinogenesis.^1^ Furthermore, rising evidence also appeared about rare potential neurological consequences such as inflammatory demyelinating polyradiculoneuropathy, Guillain-Barré syndrome, multiple sclerosis, and neuropathy as well as occasional reports of seizures.^2^

Posterior reversible encephalopathy syndrome (PRES) is a rare but serious neurological condition that has been reported in fewer than 10 cases. It manifests with symptoms such as headaches, nausea/vomiting, seizure, and other focal neurological deficits and is diagnosed based on specific clinical and radiological features.^3^ PRES is usually triggered by high blood pressure, cytotoxic agents, renal failure, immunosuppressive therapy, and some autoimmune conditions.^4,5^ We present a case of PRES that occurred after infliximab treatment in a patient with fistulizing CD. In this report, infliximab is being discussed as a potential trigger for PRES in a patient with fistulizing CD and adds to the limited body of knowledge on this rare neurological complication.

## CASE REPORT

A 19-year-old woman with an unremarkable medical history was diagnosed with CD 4 months before admission. She presented to our hospital with fistulizing CD and peri-iliac abscess, for which she required biologic therapy, so she was given infliximab. The patient was diagnosed on December 1, 2022, via a computed tomography (CT) scan, which revealed features of CD with a peri-ileal abscess. Therefore, treatment with intravenous antibiotics was initiated for 14 days, before an endoscopy was performed, which showed an edematous ileocecal valve, ulceration, and narrowing of the right colon that could not be passed by a scope, with a normal anal canal and left colon, in which the diagnosis of CD was confirmed by histopathology. Following successful management of the abscess, the patient was started on adalimumab with tapering prednisolone for biological treatment of CD on the January 1, 2023; however, her treatment was interrupted many times.

**Figure 1.**
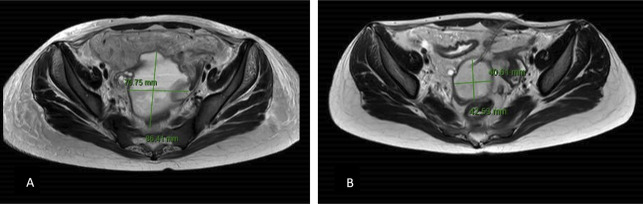
(A) Pelvic MRI showed a large pelvic fluid collection in the Douglas pouch measuring 7 × 9 × 4 cm (left-right × anterior-posterior × cranial-caudal), not separated from the sigmoid colon, associated with diffuse surrounding fat creeping and edema. (B) 1 week after the drain was inserted, showing a slight decrease in size. MRI, magnetic resonance imaging.

Upon admission to the hospital in February 2023, the patient presented with a severe flare of CD, manifested by increasing crampy abdominal pain, watery diarrhea, and nonprojectile vomiting. Pelvic magnetic resonance imaging (MRI) showed features of CD complicated with a complex fistula, connected to the terminal ileum and had a peri-ileal abscess approximately 7 × 9 × 4 cm left-right, anterior-posterior, cranial-caudal (Figure 1). As a result, a decision was made to administer an infliximab infusion (300 mg), along with intravenous antibiotics for the abscess, with a low dose of methylprednisolone steroid. After a thorough assessment of the patient's clinical condition and extensive discussions with the surgical team, the decision was not to perform abscess drainage before initiating infliximab therapy. Despite the presence of an abscess, its stability, with a lack of significant symptoms or complications at the time of admission, and no considerable amount of fluid contributed to the decision to prioritize the initiation of infliximab therapy with no need for urgent surgical intervention.

Moreover, initiating infliximab treatment before abscess drainage was further validated by existing evidence. For example, Cullen et al conducted a retrospective study involving 13 CD patients with phlegmons, all of whom received antibiotics. Most patients experienced resolution or improvement with anti-TNF therapy, with only a minority requiring subsequent surgery. Notably, the use of anti-TNF therapy did not appear to elevate the risk of infectious complications, underscoring its favorable safety profile in this scenario.^6,7^

**Figure 2.**
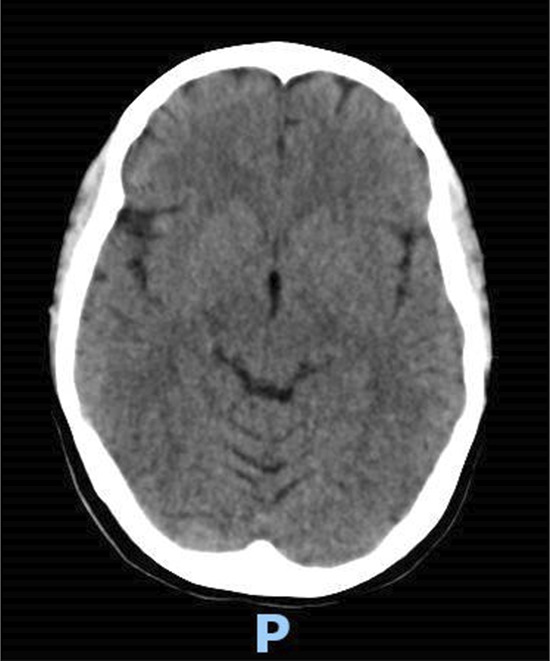
Brain CT scan without contrast showed no hemorrhage; normal brain CT. CT, computed tomography.

**Figure 3.**
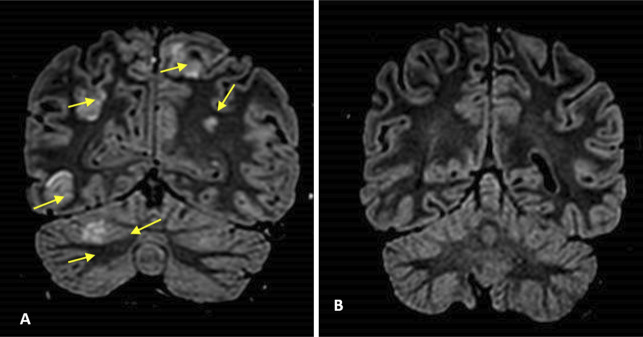
(A) Brain magnetic resonance imaging showed multiple bilateral less or more symmetrical cortical FLAIR hyperintensities (arrows) involving both occipital, posterior temporal, left parietal, and frontal lobes with scattered foci of enhancement. (B) Resolved previously noted multiple bilateral cortical T2/FLAIR hyperintensities involving both occipital. FLAIR, fluid-attenuated inversion recovery.

Eight days following the first infliximab dose, the patient developed signs and symptoms of increased intracranial pressure, defined as a severe headache that was associated with nausea, vomiting, and high blood pressure readings exceeding 160/90 mm Hg. A CT scan without contrast was performed promptly and rolled out hemorrhage (Figure 2). Moreover, 5 hours later, she experienced tonic-clonic convulsions, despite receiving intravenous benzodiazepines and phenytoin. She had 3 consecutive episodes and failed to regain her baseline level of consciousness, requiring urgent intubation and admission to the intensive care unit. A brain MRI showed multiple bilateral symmetrical cortical T2/fluid-attenuated inversion recovery hyperintensities affecting both occipital, posterior temporal, left parietal, and frontal lobes with scattered foci of enhancement along with mild subarachnoid hemorrhage (Figure 3). A lumbar puncture was performed, and the cerebrospinal fluid analysis came back normal. Given the clinical presentation and the MRI findings, the diagnosis of PRES was established. The patient was extubated the following day and started on oral levetiracetam and phenytoin as advised by the neurologist.

Following the diagnosis of PRES, the patient did not experience any further seizures, had stable blood pressure readings without the need for antihypertensive medications, had no neurological deficits, and her intellectual function remained normal. Fourteen days after the first dose of infliximab and 9 days after the PRES diagnosis, it was decided to give the second dose of infliximab (300 mg) along with intravenous methylprednisolone that was already commenced, then was tapered off. There were no immediate complications after the administration of infliximab. However, 10 days after the second dose, the patient's blood pressure readings began to rise and she complained of headache and vomiting. Hence, the patient was immediately managed with intravenous labetalol and underwent brain MRI, which showed resolution of previously noted multiple bilateral cortical T2/fluid-attenuated inversion recovery hyperintensities (Figure 3). The patient had no seizures or neurological deficits, and her symptoms resolved a day later. The patient's blood pressure was controlled with low-dose amlodipine.

After the patient's condition had stabilized, she received intravenous antibiotics for approximately 2 weeks and underwent a CT-guided drainage of the intra-abdominal abscess 2 weeks after admission, with a drain left in place. The timing of CT-guided drainage following biologic treatment was determined based on the patient's individualized clinical condition, characterized by severe CD and evidence of ongoing sepsis with elevated inflammatory markers. Although performing drainage before initiating infliximab therapy could theoretically reduce the risk of sepsis, the initial decision was to proceed with infliximab to avoid surgical intervention, as the abscess was deemed nonurgent, aligning with previous evidence. However, 2 weeks later, despite intravenous antibiotics, the patient's condition did not improve, with inflammatory markers continuing to rise and persistent fever. Subsequently, a multidisciplinary discussion involving the gastroenterologist, surgeon, and anesthesia team led to the decision to proceed with drainage, which came after the first dose of infliximab.

One week later, a follow-up MRI showed a decrease in abscess size (Figure 1). Approximately 4 weeks after admission, the drain was removed after it stopped functioning, and an abdominal ultrasound showed complete resolution of the abscess. Both abcess resolution and the patient's negative inflammatory marker results allowed for a switch to oral antibiotics for 7 days. During this time, an ileostomy was created as part of the plan for a future colectomy to rest the colon. The decision to proceed with ileostomy as a precursor to colectomy was made collaboratively with gastroenterologists and surgeons, drawing upon a comprehensive assessment of the patient's disease severity and treatment response, due to the extensive disease involvement as having a severe Crohn's flare, which necessitated prompt and aggressive management to control disease activity and prevent potential complications, further emphasizing the urgency of effective treatment strategies and the necessity for definitive management. Moreover, other biologic options were considered; however, they were not feasible for this patient as they were unavailable in the patient's region. The patient experienced significant weight loss and was kept on total parenteral nutrition until she was able to resume a soft to regular diet.

The patient received the third dose of infliximab 4 weeks after the second dose and was discharged in a generally good condition 2 days later. She continued on oral levetiracetam with no antihypertensive medications needed. Follow-up appointments were scheduled for 1 week and 2 weeks after discharge, and there were no complications reported.

## DISCUSSION

The exact cause of PRES is not fully understood. Two theories proposed by Bartynski are that hypertension leads to hyperperfusion, destroying autoregulation and damaging the blood-brain barrier that subsequently causes vasogenic edema. The other theory is a toxic effect, causing endothelial dysfunction and vasoconstriction that subsequently leads to edema.^8^ In such a way, infliximab, which binds to TNF-alpha, by antibody-dependent cellular toxicity or complement-dependent cytotoxicity, can lead to cell destruction and edema, which could be the mechanism behind PRES.^9^ In this case, hypertension was noted as the first manifestation, supporting the first theory. However, in other studies, PRES developed without preceding hypertension.^9^

In the literature, very few cases described infliximab-induced PRES. One case, reported by Haddock et al, involved an 8-year-old girl who was given infliximab for CD and underwent laparotomy and colectomy. Thirteen days after receiving the infliximab dose, the patient had a tonic-clonic seizure that required intubation. The patient experienced 3 seizure episodes in a short period managed by levetiracetam and midazolam, with an MRI finding diagnosing PRES, for which her CD was maintained on azathioprine, while infliximab was not given again. The patient was followed for 2 years with no recurrence of seizures.^9^ In another case, a 24-year-old woman with fistulizing CD received infliximab twice, with the doses being 7 days apart. She developed PRES 3 days after the second dose, with a presentation similar to that of our case, as she had developed signs of increased intracranial pressure, followed by 2 episodes of generalized tonic-clonic seizures. The patient improved after a week and was kept on immunomodulatory therapy.^10^

A third similar case by Zamvar et al was a 14-year-old adolescent girl with CD who experienced several seizure episodes 5 days after receiving infliximab. An MRI revealed the diagnosis of PRES, which was managed with phenytoin. She was later discharged on azathioprine, and a repeat MRI showed complete resolution 4 weeks later, with no recurrence of seizure. However, the patient experienced CD flare later on, as infliximab was not given once more, which needed colectomy and ileostomy.^11^ These 3 cases depict a scenario of PRES after infliximab, with similar presentations to the current case, including initial signs of raised intracranial pressure followed by seizure episodes, and typical MRI findings of soft white matter vasogenic edema in the posterior cerebral hemispheres, in the parieto-occipital region in particular.

The timing of PRES occurrence after infliximab treatment varied in the literature, with our case occurring after 8 days, which is in range to other reports, as was 5 days in the report presented by Haddock et al and 13 days in that by Zamvar et al.^9,11^ None of these reports tried to resume infliximab after, in contrast to our practice, as the second and third dose was given as scheduled 2 and 7 weeks after the first dose, respectively, with a follow-up MRI showing resolution of the MRI lesions, and the patient had no recurrence of seizure in the 8-week follow-up. This entails that this process may not be recurrent with the same triggering agent, the immediate management of blood pressure may have prevented PRES from developing, or perhaps another process played a role in the patient's improved outcome. Moreover, our rationale for the decision to resume infliximab therapy after the initial episode of PRES was made following careful evaluation and consultation with a multidisciplinary team of gastroenterologists, neurologists, and surgeons and was based on a comprehensive risk-benefit analysis, weighing the potential benefits of infliximab in managing the patient's severe CD against the risk of PRES recurrence. Despite the recurrence of symptoms following the second dose, the patient exhibited notable improvement with supportive management, especially since other biological therapies were not available in Palestine during that period, making infliximab the only option. Keeping the patient with no biological treatment, the sequences would be far greater than the potential for PRES emergence. Furthermore, the absence of immediate complications after the third dose, with sustained resolution of symptoms and the absence of recurrence during follow-up, provided evidence supporting the continuation of infliximab therapy. In addition, it is crucial to consider the patient's preference for infliximab therapy, as they expressed a strong desire to continue with a treatment that they perceived as effective and well tolerated. Moreover, the gastroenterologist's previous experiences with infliximab in similar cases, with rare side effects, contributed to the decision to maintain infliximab therapy. The need for ileostomy as a precursor for colectomy is also supported by previous evidence; according to a 2022 meta-analysis, it was found that patients who underwent ileocecal resection had lower recurrence rates compared with those receiving biological therapy. The odds ratio for clinical recurrence with biologics vs postsurgical treatment was 2.50 (95% confidence interval 1.53–4.08, *P* < 0.001).^12^

There is no definitive treatment of PRES; management relies on early diagnosis, supportive measures, such as adequate hydration and electrolyte replacement, stopping the agent that caused it or decreasing the dose, controlling blood pressure, and seizure management.^5,10^ Generally, PRES is a benign condition with a favorable prognosis, mortality is observed in 19% of cases, and it ends in functional impairments in approximately 44% of patients.^5^

In conclusion, PRES is a rare, serious potential side effect of infliximab, but reversible, that can be managed and prevented with early recognition by supportive care, seizure management, and blood pressure control. Nonetheless, additional extended-term research is necessary to investigate the condition further.

## DISCLOSURES

Author contributions: R. Rabi and Q. Abdoh contributed to the work reported, study design and execution, and acquisition of data regarding the case. R. Rabi and B. Musmar took part in writing and discussion. R. Rabi, A. Abuhassan, A. Barqawi, Q. Abdoh took part in revising the article and approving it for publication. R. Rabi is the article guarantor.

Acknowledgments: We would like to thank all teams involved in the management of the case for providing detailed information about the case and had contributions in facilitating the data collection.

Financial disclosure: None to report.

Statement of ethics: Ethical approval is not required for this study in accordance with local or national guidelines, according to An Najah National University Ethics Committee.

Informed consent was obtained for this case report.

Data availability statements: All data generated or analyzed during this study are included in this article. Further are available upon request to the corresponding author.
